# Extradural Lumbar Spinal Angiolipoma: A Case Report and Review of Literature on Rare Benign Tumors

**DOI:** 10.7759/cureus.103764

**Published:** 2026-02-17

**Authors:** Georges Jammal, Joseph Akiki, Carole Kesrouani, Ronald Moussa, Gilles El Hage

**Affiliations:** 1 Department of Neurosurgery, Faculty of Medicine, Hôtel Dieu de France Hospital, Saint Joseph University of Beirut, Beirut, LBN; 2 Department of Pathology, Faculty of Medicine, Hôtel Dieu de France Hospital, Saint Joseph University of Beirut, Beirut, LBN

**Keywords:** epidural lesion, extradural tumor, lumbar spine, rare tumors, spinal angiolipoma

## Abstract

Spinal angiolipomas (SALs) are rare benign tumors composed of mature adipose tissue interspersed with abnormal vascular channels. Their occurrence in the lumbar region is particularly exceptional, with fewer than 20 cases reported in the literature. The clinical presentation is often nonspecific and typically reflects chronic spinal cord or nerve root compression, which can lead to delayed diagnosis. We report the case of a 58-year-old woman who presented with progressive lower back pain persisting for 18 months and initially attributed to musculoskeletal causes. Magnetic resonance imaging (MRI) revealed a well-circumscribed lesion at the L4 level showing both fatty and vascular components, while computed tomography confirmed the absence of calcification or bone erosion. The patient underwent complete resection through hemilaminectomy, which achieved total removal of the lesion. Histopathological examination demonstrated a nonencapsulated angiolipoma without malignant features. Postoperative recovery was uneventful, with resolution of symptoms and no evidence of recurrence on follow-up. This case highlights the diagnostic challenges posed by lumbar SALs, emphasizes the pivotal role of MRI in differentiating them from other epidural masses, and underlines the excellent prognosis achieved with surgical excision. Given their benign nature and lack of malignant potential, clinical awareness and accurate radiologic interpretation remain key to ensuring appropriate management when faced with unexplained chronic lumbar pain.

## Introduction

Angiolipomas are typically benign tumors that may be found in various regions such as the neck, trunk, and forearm, often presenting subtly [1]. While angiolipomas are most commonly nontender lesions located on the trunk and upper extremities, including rare instances on the palms and digits, digital angiolipomas are typically painful [2]. Other unusual forms, such as spinal angiolipomas (SALs), exhibit distinct clinical and pathological characteristics.

SALs are benign tumors composed of mature adipose tissue interspersed with abnormal vascular channels [3]. They are exceedingly rare, accounting for only 0.04%-1.2% of all tumors affecting the spinal axis. However, within the epidural space, SALs represent 2%-3% of all tumors in that location [4,5]. Although benign, SALs can occasionally infiltrate adjacent structures [6].

SALs were first described in the early literature and remain rare benign tumors of the spinal epidural space. These tumors demonstrate a female predominance and typically present in patients during the fourth and fifth decades of life [7].

Most SALs are located in the thoracic region of the spinal cord, particularly between T2 and T5, and can arise in both intradural and extradural forms [8,9]. In contrast, lumbar angiolipomas are exceptionally uncommon, comprising only a small fraction of all spinal extradural angiolipomas [10].

Before diagnosis through magnetic resonance imaging (MRI), patients with SALs often present with nonspecific symptoms related to spinal cord or nerve root compression. These symptoms may include back pain, lower extremity weakness, sensory disturbances, hyperreflexia, spasticity, and sphincter dysfunction [11]. The progression of symptoms is typically gradual due to slow tumor growth and compression, but can occasionally present acutely in cases of intratumoral hemorrhage or venous thrombosis [12]. The duration of symptoms before diagnosis can vary widely, ranging from 1 to 180 months.

Total surgical resection is considered the treatment of choice, with laminectomy alone being sufficient in most cases to achieve complete tumor removal and symptom relief [12]. In this paper, we present a new case of extradural lumbar SAL and discuss the relevant literature to emphasize its rarity, clinical features, and management considerations.

## Case presentation

**Figure 1:**
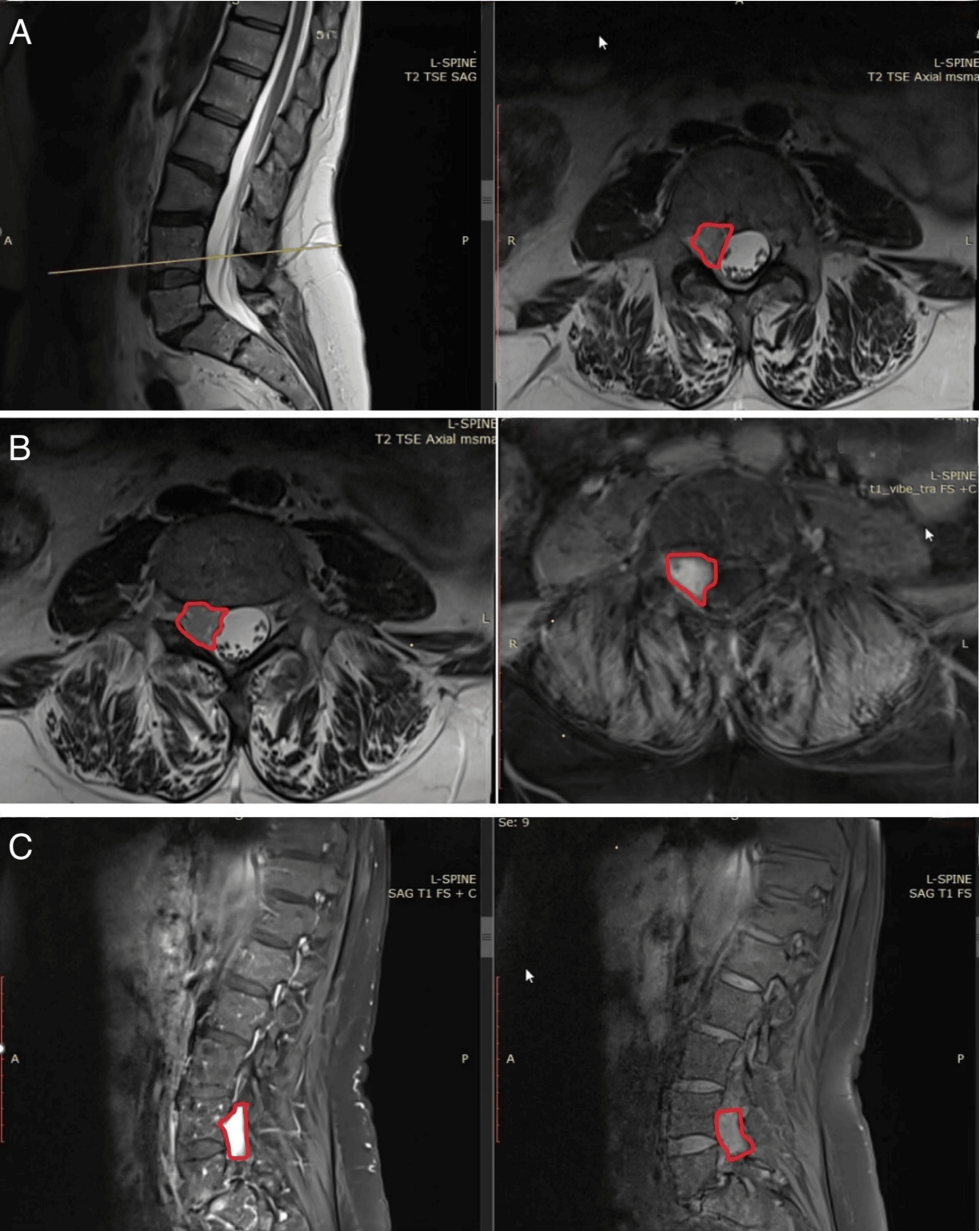
Magnetic resonance imaging of the lumbar spine showing an extradural lesion at the L4-L5 level (A) Sagittal (left) and axial (right) T2-weighted images demonstrating a well-circumscribed extradural lesion (outlined in red) located posterior to the L4 vertebral body, causing slight compression of the dural sac and the contained nerve roots. (B) Axial T2-weighted (left) and T1-weighted postcontrast (right) sequences showing the same lesion with intermediate signal intensity on T2 and intense enhancement after gadolinium injection, consistent with an extradural mass. (C) Sagittal T1-weighted (right) and T1-weighted postcontrast (left) images confirming homogeneous contrast enhancement of the lesion, better delineating its extradural location and its relationship with surrounding structures

A 58-year-old woman was admitted to our department with severe lower back pain that had persisted and progressively worsened over 18 months. She denied any radiation of pain to the lower limbs, as well as motor deficits or sphincter dysfunction. Prior to admission, the patient had undergone a comprehensive clinical and radiological evaluation. MRI of the lumbar spine revealed a hyperintense, well-defined extradural lesion located at the L4 level (Figure 1).

A lumbosacral computed tomography (CT) scan was also performed to assess the bony structures in relation to the lesion. The CT scan confirmed the presence of an intracanal, extradural soft tissue mass located on the right lateral aspect of the spinal canal at the L4 level, without evidence of intralesional calcifications or adjacent bony erosion.

**Figure 2:**
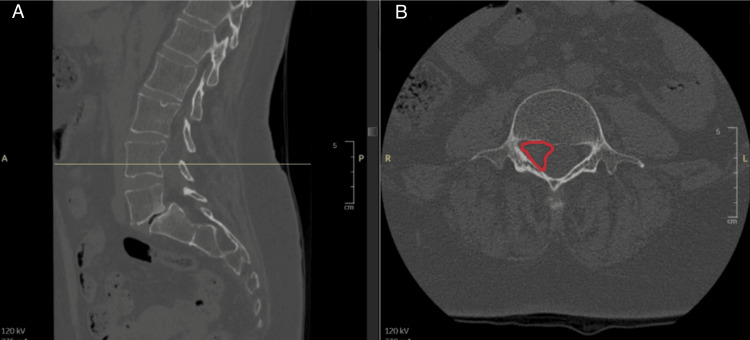
Lumbosacral CT scan demonstrating an intradural extramedullary lesion in the L4-L5 region (A) Sagittal CT (bone window) with the reference line indicating the level of the axial image. (B) Axial CT (bone window) at the L4 vertebral body level showing an intradural extramedullary lesion (highlighted) CT: computed tomography

Additionally, at the L5-S1 level, a grade I spondylolisthesis of L5 was noted, secondary to bilateral isthmic lysis, accompanied by disc degeneration resulting in relative narrowing of the foramina (Figure 2).

Upon admission, the patient was conscious, cooperative, and well-oriented, with no known comorbidities or allergies. Neurological examination of the lower limbs revealed preserved motor strength (grade 5/5 bilaterally in both proximal and distal muscle groups, as assessed using the Medical Research Council scale). Superficial and deep sensory modalities were intact bilaterally. Deep tendon reflexes were symmetrical and normoactive (2+) at the patellar and Achilles tendons. Plantar responses were flexor bilaterally (Babinski negative), with no pyramidal signs. Gait examination was unremarkable, and no sphincter dysfunction was observed.

**Figure 3:**
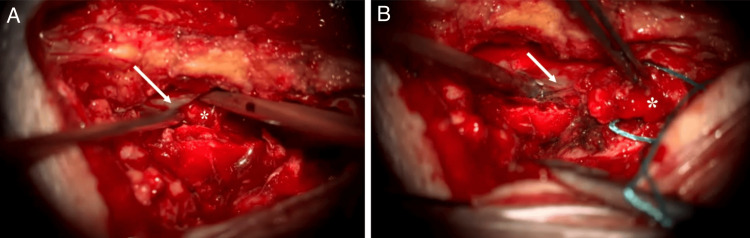
Intraoperative views showing exposure and resection of the extradural lesion (A) Following right hemilaminectomy of L4 and hemilaminotomy of L3, the dural sac (arrow) is exposed, and an extradural mass (asterisk) is visualized beneath it. (B) The extradural lesion (asterisk) is carefully dissected and separated from the dural sac (arrow) under microscopic visualization. The white arrow shows the dural sheath, and the asterisk indicates the spinal tumor

Surgical intervention was carried out under general anesthesia, with the patient positioned prone on horizontal bolsters. A right L4 hemilaminectomy combined with a hemilaminotomy of L3 was performed, allowing for complete resection of the extradural lesion without dural breach. The excised tissue was subsequently sent for histopathological examination (Figure 3).

Postoperatively, the patient was transferred to the ward without complications. A surgical drain was placed, and the wound dressing remained clean and dry throughout her stay. On postoperative day (POD)1, the patient mobilized for the first time, exhibiting no neurological deficits. A mild limitation in hip flexion was noted, attributed to localized pain at the surgical site. The drain was removed on POD1, having collected a total output of 10 cc. The patient was discharged on POD2 in stable condition, with no neurological deficits, pain, or paresthesia.

**Figure 4:**
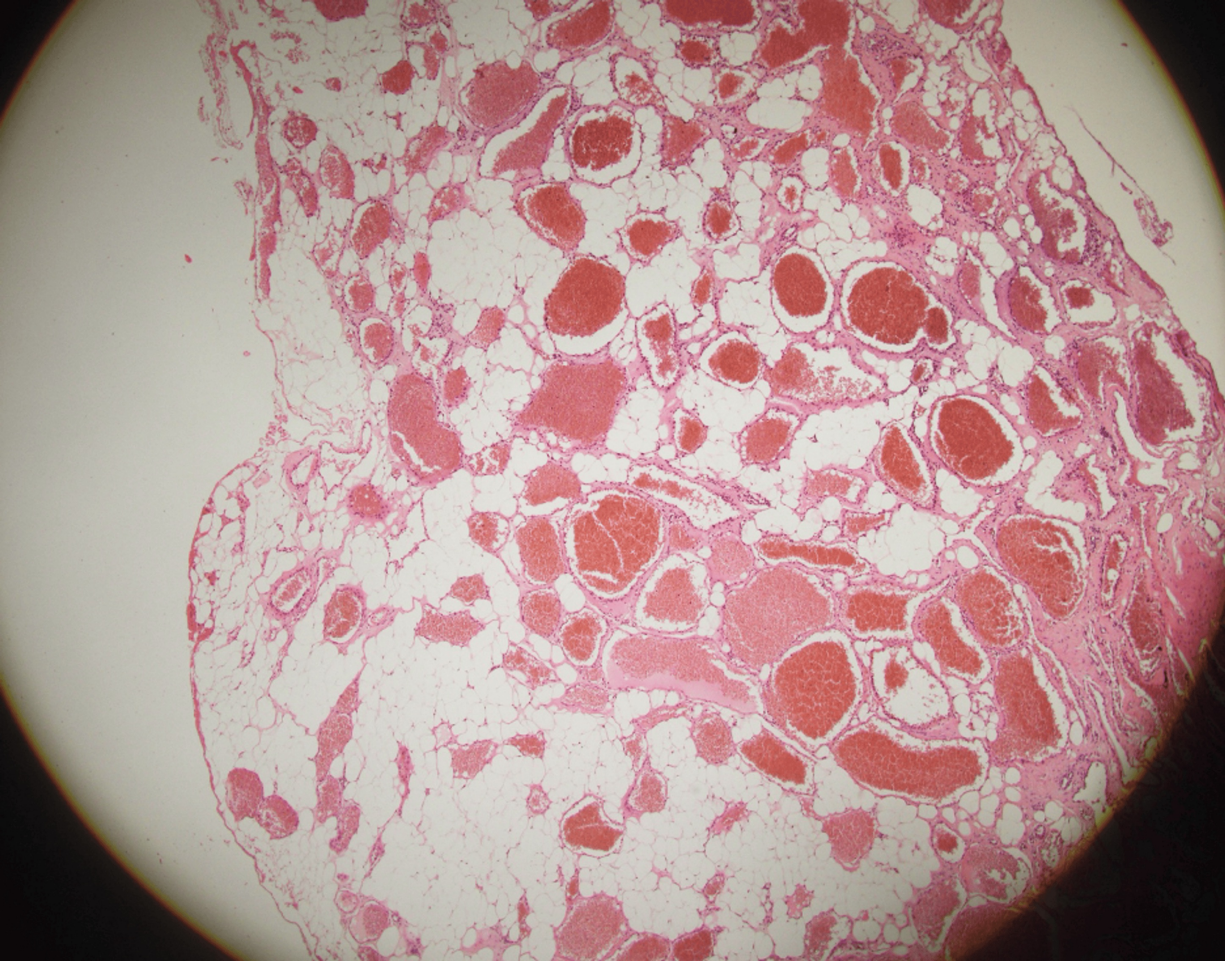
A nonencapsulated lobulated lesion, alternating two components, adipose and vascular

**Figure 5:**
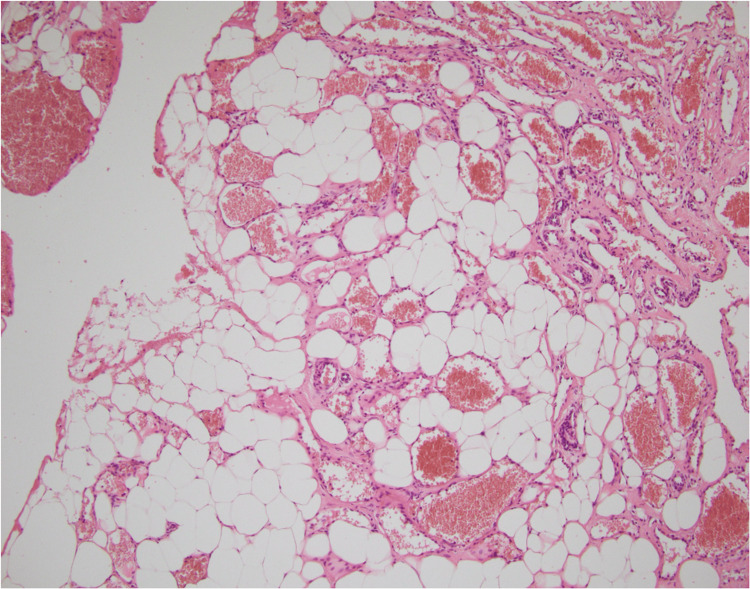
Sheets of regular adipocytes associated with vascular structures of variable caliber

Histopathological examination of the excised lesion showed a vaguely lobulated tumor proliferation, nonencapsulated at the periphery. It consists of two alternating components of relatively proportional distribution (Figure 4). The first component consists of sheets and nests of regular adipocyte cells with a regular eccentric nucleus without cytonuclear atypia. The second component consists of vascular structures of variable size, small to medium, and sometimes large, gaping (Figure 5). They are often congested; some contain endoluminal thrombi with recanalization, while others are filled with fibrinous material. They are lined with a flattened or turgid endothelium without cytonuclear atypia. They are most often thin-walled, without constituted muscle fibers. Immunohistochemical studies reveal diffuse CD34 positivity within the vascular structures, with positivity in certain sectors for CD31.

Follow-up

The patient presented with a grade I spondylolisthesis without evidence of instability on dynamic radiographs. As the angiolipoma was located at a level superior to the spondylolisthesis, no surgical intervention was undertaken for the spondylolisthesis. Postoperatively, the patient underwent physiotherapy focusing on muscular strengthening. Follow-up evaluations at one and six months demonstrated excellent clinical outcomes, with complete resolution of pain. At the one-year follow-up, the patient remained pain-free, without spinal tenderness or neurological deficits. The favorable postoperative course suggests that the patient’s symptoms were caused by the angiolipoma rather than the spondylolisthesis, given that only the angiolipoma was resected and durable symptom resolution was achieved.

## Discussion

SALs are rare benign tumors that account for a very small percentage of spinal epidural lesions. They are most frequently observed in women aged 40-60 years, although reported cases range from 1.5 to 85 years [13]. While SALs are most commonly located in the thoracic spine [3], lumbar localization, as seen in our case, is particularly rare, with only about 20 cases described in the literature. This rarity often leads to alternative diagnoses being considered initially.

Clinically, SALs typically present with nonspecific symptoms related to spinal cord or nerve root compression, including sensory disturbances, motor deficits, and progressive weakness, depending on the location and size of the lesion [14]. The slow-growing nature of these tumors often leads to delayed diagnosis, with many cases identified only after more than a year of symptom progression. Our patient experienced severe lower back pain with progressive worsening over 18 months, which aligns with previous reports of prolonged diagnostic delays. Acute neurological deterioration can occur if there is rapid tumor growth due to thrombosis or hemorrhage within the lesion [3].

Histopathologically, SALs are characterized by a mixture of mature adipose tissue and abnormal vascular components, with the fat-to-vessel ratio typically ranging from 1:3 to 2:3 [6-15]. Importantly, these lesions lack atypia, pleomorphism, and mitotic figures [16], supporting their benign nature. Although many SALs are surrounded by a thin capsule [5], our case revealed a nonencapsulated angiolipoma without malignant features.

MRI remains the gold standard for diagnosis, often revealing a spindle-shaped lesion adherent to the dura mater, displacement of the spinal cord (the "noodle sign"), and specific signal characteristics due to the mixed fat and vascular content [17]. Typically, the adipose component appears hyperintense on both T1- and T2-weighted images and hypointense on fat-suppressed sequences, while vascular components enhance vividly after gadolinium administration. These features are critical for differentiating SALs from other spinal lesions, such as lipomas, vascular malformations, meningiomas, metastases, and hematologic malignancies.

CT may demonstrate hypodense lesions relative to the spinal cord, and although features like trabeculation, calcification, and bony erosion have been reported [18,19], our patient’s CT scan showed a right lateral extradural mass at L4 without calcification or bony changes. Spinal X-rays are often unremarkable but may reveal erosion of vertebral bodies and pedicles in cases of infiltrative growth [13].

The patient presented with isolated lower back pain, without signs of radiculopathy. This highlights the importance of carefully considering such symptoms and avoiding reliance on symptomatic treatment without imaging. In cases like this, where the lesion is benign and lacks malignant potential even if left untreated for years, the emphasis shifts from urgency to precision. Detailed imaging becomes essential not only for early detection but also for identifying subtle features that may explain the clinical presentation. This case reinforces the need to carefully evaluate every imaging detail, as it may reveal the true underlying pathology.

The treatment of choice for both infiltrating and noninfiltrating SALs is total surgical resection, which typically leads to excellent outcomes [20]. In our case, a posterior approach enabled complete removal of the tumor, resulting in symptom resolution. Given the benign nature of SALs, recurrence is uncommon but may occur, particularly if complete resection is not achieved due to dural infiltration [3].

To better situate our case within the existing body of evidence, we reviewed the literature on SALs and summarized the key findings in (Table 1). This comparative overview highlights the variability in clinical presentation, imaging features, treatment approaches, and postoperative outcomes across reported cases.

**Table 1:** Comparative summary of reported cases of SALs in the literature NI: no information; DDx: differential diagnosis; MRI: magnetic resonance imaging; CT: computed tomography; SAL: spinal angiolipoma

Study	Type	n (cases)	Predominant location	Age/sex	Clinical presentation	Imaging features	Treatment	Outcome
Si et al. [5]	Retrospective series	21	Thoracic > lumbar	Adults, middle-aged; female predominance	Back pain, progressive cord/root compression; delayed diagnosis common	MRI: T1/T2 hyperintense fatty component, vascular part enhances after gadolinium; classification proposed (intraspinal vs. dumbbell, infiltrating vs. noninfiltrating)	Surgical resection	Excellent prognosis with total excision; recurrence rare
Wang and Tang [3]	2 case reports + review	2	One thoracic, one lumbar	Adults	Back pain, weakness	MRI: fat + vascular enhancement	Total surgical excision	Full recovery, no recurrence
Trabulo et al. [16]	2 cases + review	~60 cases reviewed	Thoracic predominant	Wide age range (children to elderly)	Chronic compression symptoms	CT/MRI: extradural, fatty/vascular mix; infiltrating vs. encapsulated	Surgical excision	Benign, recurrence rare unless dural infiltration
Haddad et al. [15]	2 cases + review	2 + literature	Extradural	Adults	Pain, radiculopathy	Imaging and histopathology	Surgical removal	Good post-op outcomes
Rocchi et al. [17]	2 cases (lumbar) + review	2	Lumbar (rare)	Adults	Low back pain	MRI: mixed fat/vascular mass; mimics lipoma, meningioma, metastasis	Posterior resection	Full recovery
Kang et al. [20]	Case report	1	Lumbar	Adult	Progressive low back pain	MRI confirmed SAL features	Resection	Complete recovery
Weill et al. [18]	Imaging series	NI	Various	NI	NI	CT: hypodense lesions; MRI: fat hyperintensity + vascular enhancement	NI	NI
Apostolakis et al. [6]	Review (2012-2017 cases)	Literature synthesis	Thoracic predominant	NI	Pain, compression	Summarized MRI/CT features; emphasized DDx	Surgery	Good outcomes

In summary, although SALs are rare and can present diagnostic challenges due to their nonspecific symptoms and unusual location, awareness of their imaging and histopathological features is critical for timely diagnosis and effective treatment. Surgical resection remains curative in most cases, with a favorable prognosis when total excision is accomplished.

## Conclusions

This case highlights the exceptional rarity of lumbar SALs and the importance of maintaining a broad diagnostic perspective when evaluating patients with chronic back pain and progressive neurological symptoms. Clinicians should take patients’ complaints seriously and avoid converging too quickly on common diagnoses, as this may delay the identification of rarer but clinically significant pathologies. Early recognition relies on careful clinical evaluation supported by appropriate imaging, particularly MRI, which remains crucial for accurate characterization and surgical planning. Complete surgical excision continues to offer excellent long-term outcomes, as reflected in our patient’s full recovery and absence of recurrence. Ultimately, heightened awareness of this rare entity and a vigilant, symptom-driven approach can help ensure timely diagnosis, effective management, and the prevention of irreversible neurological deficits.
